# Preferences of patients for benefits and risks of insomnia medications using data elicited during two phase III clinical trials

**DOI:** 10.1093/sleep/zsac204

**Published:** 2022-09-02

**Authors:** Sebastian Heidenreich, Melissa Ross, Gin Nie Chua, Dalma Seboek Kinter, Andrea Phillips-Beyer

**Affiliations:** Patient Centered Research, Evidera, London, UK; Patient Centered Research, Evidera, Bethesda, MD, USA; Patient Centered Research, Evidera, London, UK; Idorsia, Allschwil, Switzerland; Innovus Consulting, London, UK

**Keywords:** insomnia, patient preferences, discrete choice experiment, clinical trial, phase III

## Abstract

**Study Objectives:**

To elicit the trade-offs patients are willing to make between benefits and risks of medications for chronic insomnia, with the purpose of allowing a patient-centric interpretation of clinical trial data.

**Methods:**

A discrete choice experiment (DCE) was included in the two placebo-controlled phase III trials that evaluated the efficacy and safety of daridorexant. The DCE design was informed by a two-phase qualitative study, followed by qualitative and quantitative pilot testing before fielding. Relative attribute importance (RAI) and acceptable trade-offs between benefits and risks were obtained using a mixed logit model.

**Results:**

Preferences were elicited from 602 trial participants (68.1% female, aged 58.6 ± 14.5 years). Preferences were most affected by daytime functioning (RAI = 33.7%) as a treatment benefit and withdrawal symptoms (RAI = 27.5%) as a risk. Patients also valued shorter sleep onset (RAI = 6.4%), longer sleep maintenance (RAI = 5.4%), reduced likelihood of abnormal thoughts and behavioral changes (RAI = 11.3%), reduced likelihood of dizziness/grogginess (RAI = 9.2%), and reduced likelihood of falls at night (RAI = 6.5%). Patients were willing to make trade-offs between these attributes. For example, they would accept an additional 18.8% risk of abnormal thoughts and behavioral changes to improve their daytime functioning from difficult to restricted and an additional 8.1% risk of abnormal thoughts and behavioral changes to avoid moderate withdrawal effects.

**Conclusions:**

Patients with insomnia were willing to make trade-offs between multiple benefits and risks of pharmacological treatments. Because patients valued daytime functioning more than sleep latency and duration, we recommend that functional outcomes and sleep quality be considered in treatment development and evaluation.

## Graphical Abstract

**Graphical Abstract:**
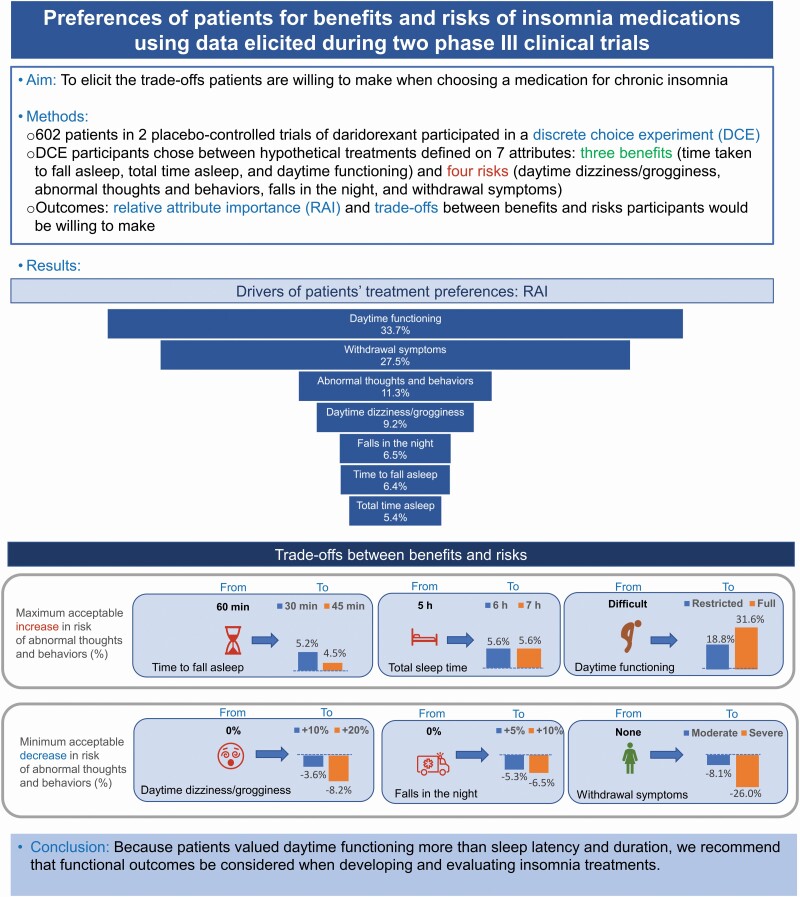

**Statement of Significance**

Little is known about the preferences of patients with insomnia for pharmacological treatments or the trade-offs between benefits and risks they are willing to make. This study showed that patients’ preferences for insomnia medications are strongly driven by a desire to improve daytime functioning and avoid medication withdrawal effects. This supports using daytime functioning as an endpoint when evaluating the efficacy of insomnia treatments and carefully weighing the efficacy of a treatment in improving daytime functioning against its risks when making a treatment recommendation in medical practice. The results of this study allow for a patient-centric interpretation of clinical trial data.

## Introduction

Several medications with varying safety and efficacy are currently approved for the treatment of chronic insomnia [1]. For example, benzodiazepines and Z-drugs, such as zolpidem, can decrease sleep onset time, reduce wake time after sleep onset, and improve sleep duration and quality. However, they can also cause drowsiness, dizziness, cognitive impairment, dependence, and other adverse effects [2–7]. Recently, a boxed warning was added to the US labeling for zolpidem, noting that its use is associated with complex sleep behaviors [8, 9]. Dual orexin receptor antagonists, developed over the last two decades, have advanced the pharmacological management of insomnia because they improve sleep outcomes but do not appear to lead to dependence or tolerance, although they may cause somnolence or fatigue [10]. For example, daridorexant was tested in two large, phase III, multi-center, double-blinded, randomized, placebo-controlled studies conducted in adult and elderly patients with insomnia disorder [11]. The two trials showed improved sleep outcomes in adults with insomnia treated with daridorexant 25 mg and 50 mg and improved daytime functioning in those treated with daridorexant 50 mg. The most common adverse effects, as with other dual orexin receptor antagonists [10], were headache, somnolence, and fatigue. Based on the results of these two trials, daridorexant was recently approved in the United States and Europe for adults with insomnia who have difficulties with sleep onset or maintenance [12]. Understanding the relative importance that patients place on various treatment aspects, such as the degree of concern with withdrawal effects associated with benzodiazepines and Z-drugs, can help guide treatment selection.

Given the complexity of the pharmacological insomnia treatment landscape, knowledge of patients’ treatment preferences, and especially the trade-offs they are willing to make between benefits and risks, can help support decision-making about specific pharmacological therapies. Additionally, understanding patients’ treatment preferences may help physicians prescribe treatments that better address patients’ needs, with the aim of improving patient satisfaction, compliance and adherence. This kind of preference information is becoming increasingly important in healthcare research in general, and it can help support the development of treatments, inform clinical guidelines, supplement health technology assessments, and contribute to regulatory decisions [13, 14]. Quantitative information about patient preferences, in particular, can facilitate patient-centric benefit-risk assessments, which can place clinical trial results in context by assessing whether patients consider the benefits of a new treatment to outweigh its risks [15].

To date, only a few studies have elicited quantitative information about treatment preferences from patients with insomnia. One study collected quantitative data on the attitudes of hospitalized patients with insomnia towards benzodiazepines versus non-pharmaceutical treatments [16]. The study found that patients’ willingness to use non-pharmaceutical treatments depended on prior exposure to benzodiazepines. Two other studies determined the willingness of patients with insomnia to pay for different aspects of insomnia treatments, including achieved outcomes, experienced adverse events, and treatment administration [17, 18]. However, the current study was the first to collect quantitative data on the benefit-risk trade-offs that patients with chronic insomnia are willing to make when choosing between alternative pharmacological treatments [10].

The results of this study make an important contribution to the literature by providing the first quantitative evidence about the benefit-risk trade-offs that patients with chronic insomnia are willing to make in the selection of pharmacological treatments. Our quantitative findings also supplement qualitative research on the perspective of patients with insomnia about their treatments [19, 20].

## Methods

### Study design

In a DCE, participants are presented with a series of tasks that ask them to choose between hypothetical treatments described by different levels of common attributes, such as benefits and risks [21, 22]. Preferences elicited from a DCE can be used to quantitatively assess how patients value the individual attributes and estimate the trade-offs they are willing to make. An unlabeled DCE was conducted as an optional sub-study among participants recruited from Germany and the US during both of the ID-078A301 (NCT03545191) and ID-078A302 (NCT03575104) multi-center placebo-controlled phase III clinical trials [11] to examine the medication preferences of patients with chronic insomnia and subsequently interpret the trial outcomes from the patients’ perspective. In the DCE, patients were asked to answer 12 choice tasks in which they had to choose between two mutually exclusive hypothetical pharmacological alternatives, each of which was described by seven benefit and risk attributes. The attribute levels were varied systematically according to an experimental design, such that respondents were required to make trade-offs when selecting their preferred treatment. The two trials were approved by the local independent ethics committee or institutional review board for each site and conducted in accordance with International Council for Harmonization Good Clinical Practice guidelines, the principles of the Declaration of Helsinki, and the laws and regulations of the respective countries in which the studies were conducted. Trial ID-078A301 was conducted between June 4, 2018 and February 25, 2020 at 75 sites in the United States, Australia, Canada, Denmark, Germany, Italy, Poland, Serbia, Spain, and Switzerland; and trial ID-078A302 was conducted between May 29, 2018 and May 14, 2020 at 81 sites in the United States, Belgium, Bulgaria, Canada, Czech Republic, Finland, France, Germany, Hungary, Republic of Korea, and Sweden. The overall design of the two trials was identical and is summarized in Supplementary Figure S1. The preference study was made available to participants in Germany and the United States.

The trials enrolled adults (≥18 years) with moderate-to-severe insomnia disorder according to Diagnostic and Statistical Manual of Mental Disorders, Fifth Edition criteria. They also had to have an Insomnia Severity Index (ISI) [23] score ≥15, evidence of difficulty with sleep onset and sleep maintenance as measured by polysomnography and recorded by the patient in a sleep diary, and a body mass index of 18.5 to 40.0 kg/m^2^. Full eligibility criteria are provided in the Supplementary Text S1. All patients had to provide signed informed consent before participation in the trial.

Patient preferences were digitally elicited using the PAtient preferences stUdy in InSomnia (PAUSe) questionnaire at treatment initiation (visit 4) and again at the end of 3 months of treatment (visit 8). The PAUSe questionnaire included a DCE, a set of brief screening questions [24–26] to assess self-reported health literacy, and five questions to assess numeracy [27]. In addition, sociodemographic and baseline clinical characteristics were collected as part of the trial.

### Attribute selection

**Figure 1.**
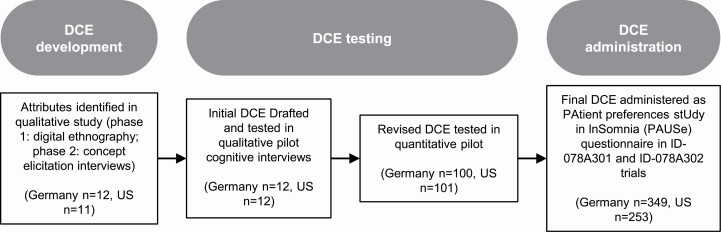
Schema of the study design. The DCE administered in the ID-078A301 and ID-078A302 trials was developed in an independent study that used an iterative process combining qualitative and quantitative approaches. Treatment attributes were identified through a dedicated two-phase qualitative concept elicitation study, which consisted of a digital ethnography and in-person concept elicitation interviews. Prior to inclusion in the trial protocols, the DCE was pre-tested in qualitative and quantitative pilots. Patient preferences were digitally elicited using the PAtient preferences stUdy in InSomnia (PAUSe) questionnaire at treatment initiation (visit 4) and again at the end of 3 months of treatment (visit 8). DCE, discrete choice experiment.

The DCE administered in the ID-078A301 and ID-078A302 trials was developed in an independent study that used an iterative process combining qualitative and quantitative approaches, in compliance with the health-preference research guidelines of the International Society for Pharmacoeconomic Outcomes Research [21] and the US Food and Drug Administration [28]. A schema of the study design is provided in Figure 1. Treatment attributes were identified through a dedicated two-phase qualitative concept elicitation study, which consisted of a digital ethnography and in-person concept elicitation interviews of 23 individuals (12 from Germany and 11 from the USA) with insomnia to ensure all included attributes were relevant to patients. The digital ethnography was conducted via an online platform in which patients were asked to upload pictures and descriptions portraying how insomnia impacted their life, as well as descriptions of the aspects of insomnia treatments that they felt were often missed or not discussed. The platform was online for 10 days before the data was downloaded and analyzed using an inductive thematic approach. Themes identified from the digital ethnography were discussed in the subsequent interviews. The one-on-one interviews were conducted in people’s homes in New York and Berlin and lasted approximately 90 min. During the interviews, participants described their history of insomnia and trade-offs they would be willing to make for desired treatment outcomes.

Previous studies demonstrated an association between insomnia patients’ treatment valuation and self-reported assessments of sleep [3, 29]. For the DCE, total time asleep and sleep onset were selected as important attributes because they were frequently endorsed concepts in the qualitative study. Daytime functioning attribute was also selected as an attribute for the DCE because participants in the qualitative study specifically endorsed daytime functioning as a perceived consequence of sleep quality.

When developing a DCE, attributes should be non-overlapping and preferentially independent. That is, the value placed on one attribute should not depend on the performance of other attributes. Therefore, although an early version of the DCE included a wake-time after sleep onset attribute, it was removed during the instrument development phase due to a perceived overlap with the daytime functioning attribute among participants in the qualitative pilot study, which prevented the independent evaluation of both endpoints. Retaining the daytime functioning attribute over the wake-time after sleep onset attribute was supported by the qualitative data and confirmed by the pilot study participants.

Adverse events of particular interest in the evaluation of medical treatments for insomnia include somnolence, complex sleep behaviors, addiction, and vertigo with associated risks of falls [11, 30–34]. These risks were frequently endorsed as concerning by qualitative study participants and are captured in the DCE as the “likelihood of daytime dizziness/grogginess”, “withdrawal symptoms”, and the “likelihood of falls in the night”.

In summary three benefits (time it takes to fall asleep, total time asleep, and daytime functioning) and four risks (likelihood of daytime dizziness/grogginess, likelihood of abnormal thoughts and behavioral changes, likelihood of falls in the night, and withdrawal symptoms) were included in the DCE based (Table 1).

**Table 1.** Attributes and levels

Attribute	Description presented to participants	Levels*
Time it takes to fall asleep	The number of minutes it takes to fall asleep	1 h^†^
		45 min
		30 min
Total time asleep	The total amount of time spent asleep during a night, from the time you fall asleep to when you are fully awake (i.e. no more sleep)	5 h^†^
		6 h
		7 h
Daytime functioning	Impairment of your next day functioning can take different forms, including: mental and physical tiredness; forgetfulness; and feeling irritable, frustrated, worried, stressed, or impatient. This impairment may make your work/school, family, and social life an effort Depending on the quantity and quality of sleep, your functioning can vary between: *Fully functioning* with only few symptoms and only to a slight degree that does not affect your daytime activities - *Restricted functioning*, with some symptoms at a degree that somewhat limits your daily activities - *Difficulty functioning*, with symptoms to a degree that prevents some of your daily activities	Difficulty functioning^†^
		Restricted functioning
		Fully functioning
Likelihood of daytime dizziness/grogginess	The number of people out of 100 who experience dizziness or grogginess in the morning or during the daytime as a hangover from treatment for sleeping problems. Symptoms may include one or more: vertigo, dizzy spells, feeling faint, wooziness or swaying, and feeling sluggish. These symptoms are different from the mental and physical tiredness that accompany poor quality of sleep. It is possible that a treatment could improve the quality and quantity of your sleep but leave you feeling groggy the next morning or during the next day	0%^†^
		10%
		20%
Likelihood of abnormal thoughts and behavioral changes	The number of people out of 100 taking the treatment who experience abnormal thoughts, behaviors, emotions, perceptions, or dreams. These might include hallucinations, delusions, abnormal dreams, including loss of dreams, nightmares, sleep talking, and sleep walking, or acting out dreams	0%^†^
		6%
		12%
Likelihood of falls in the night	The number of people out of 100 taking the treatment who experience difficulty maintaining balance and walking normally if they get up during the night, which may lead to a fall	0%^†^
		5%
		10%
Withdrawal symptoms	It is possible that those taking a treatment for sleeping problems will become dependent on it. When they stop taking the drug, they may experience withdrawal symptoms such as: irritability or anxiety, panic attacks, sensitivity to light, sound, and touch; muscle pain; headaches, pins and, needles; hand tremor, difficulty concentrating, feeling sick or faint; and a loss of appetite or memory. Withdrawal could be felt in three levels of severity: - *No withdrawal:* Not experiencing these symptoms to a noticeable degree - *Moderate withdrawal:* A few weeks or months of uncomfortable, but bearable symptoms - *Severe withdrawal:* Months, and possibly years of severe symptoms that will affect your daily life	None^†^
		Moderate
		Severe

*Attribute levels describe the possible performance of the hypothetical treatments related to each attribute.

^†^Reference level.

### Experimental design

**Figure 2.**
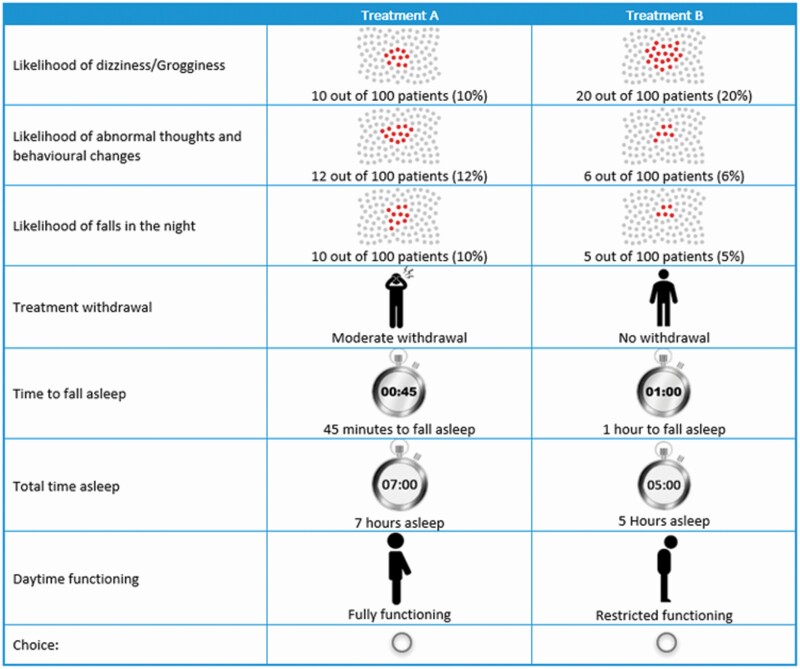
Example choice task asking patients to select one of two hypothetical treatments.

In each choice task, patients were asked to choose between two mutually exclusive unlabeled hypothetical alternatives (treatment A and treatment B), with each being described by the seven selected attributes (see Figure 2 for an example choice task). A D-efficient design was used to vary the performance of the two treatments on the different attributes across 24 choice tasks. The full set of choice tasks was split into two blocks of equal size to minimize the cognitive burden imposed on patients. Thus, patients completed one randomly assigned block of 12 randomly ordered choice tasks. Based on findings from the quantitative pilot study, treatment risks were displayed above treatment benefits in each choice task, although attributes were randomized within risks and benefits [35]. In addition to the 12 experimental choice tasks, two internal validity assessments were included: the third experimental choice task was repeated as choice task 13 to test the consistency of choices; and a dominance test with one alternative performing better on all attributes was included as choice task 14 to test patients’ engagement [36].

Before beginning the DCE, patients were provided a description of the treatment attributes and levels (Table 1). They reviewed the best and worst levels of each attribute and practiced rating their importance to familiarize themselves with the considered attributes. As a last step before beginning the DCE, patients were introduced to the format of the choice questions using a practice choice task.

### Instrument testing

Prior to inclusion in the trial protocols, the DCE was pre-tested in qualitative and quantitative pilots. First, virtual one-on-one interviews were conducted in Germany (*n* = 12) and the United States (*n* = 12) to test whether patients understood the survey tasks and could complete the DCE in line with all underlying assumptions (i.e. all relevant attributes were considered and traded) [37]. The preference survey was updated based on participant feedback in the qualitative pilot. Specifically, the amount of background information in the instructions was increased, the language was simplified, and attribute levels reworded for clarity. Second, a web-based quantitative pilot among individuals self-reporting insomnia was also conducted in Germany (*n* = 100) and the United States (*n* = 101) to validate the preference-relevance of the range covered by the levels of included DCE attributes and to select the attribute presentation that resulted in the greatest choice consistency. Details of the quantitative pilot are reported elsewhere [35]. The final survey was translated into the local languages of the included countries.

### Statistical analysis

Statistical analysis was conducted using Stata 15.1 (StataCorp LLC, College Station, TX, USA). DCE data from the 12 experimental choice tasks were analyzed within a random utility maximization framework, which assumes that in every DCE choice task, each respondent chooses the alternative that results in the highest utility (a mathematical representation of preference) of all available alternatives [38–41]. The baseline model included patients from the two phase III trials with data collected during trial visit 4 (i.e. prior to treatment initiation) as the main analysis. Data from visit 8 were analyzed separately to examine changes in preferences over the course of the trial (results not presented here). Preference data were analyzed using a dummy-coded mixed logit model, which accounts for preference heterogeneity and correlation in the data [42]. Mixed logit models with mean heterogeneity were used to compare preferences between subgroups, including trial (ID-078A301 vs. ID-078A302), visit (4 vs. 8), age (18–45 vs. 45–64 vs. ≥ 65 years), gender (male vs. female), and insomnia severity (ISI score < 22 vs. 22–28) [43].

The relative attribute importance (RAI) was calculated as the marginal utility range covered by an attribute divided by the sum of the utility ranges of each attribute. The RAI measures the proportion of changes in treatment utility that can be attributed to changes in a particular attribute.

To obtain insights into benefit-risk trade-offs, maximal acceptable risk (MAR) estimates were obtained. MAR measures the value of each attribute level, relative to its reference, in its equivalent level of risk of abnormal thoughts and behavioral changes and was included in the model as a continuous variable. Thus, MAR expressed how much additional risk of abnormal thoughts and behavioral changes patients were willing to accept for changes in other attributes. To calculate MAR, an additional mixed logit model was estimated with linear encoding of the likelihood of abnormal thoughts and behavioral changes. The linearity assumption was tested by fitting a linear function through the estimated mean marginal utilities of the likelihood of abnormal thoughts and behavioral changes and accepted for an *R*^2^ > 0.90 (observed *R*^2^ = 0.99). Further details of the statistical methods are provided in the Supplementary Text S1.

## Results

### Patients

Of the 1854 patients included in the ID-078A301 and ID-078A302 trials, 602 (32.5%) agreed to participate in the DCE sub-study. Most patients completing the DCE were female (68.1%), and the mean age was 58.6 ± 14.5 years (Table 2). Most had an ISI score of 16–21 (52.7%) and a Mini Mental State Examination score of 28–30 (94.2%). The mean body mass index (standard deviation) was 26.6 (4.4) kg/m^2^. Numeracy was considered as adequate for 87.5% of patients, and health literacy was considered as adequate for 99.5%. Patient characteristics were similar between the two trials.

**Table 2.** Patient characteristics

Characteristic	Overall sample	Trial ID-078A301	Trial ID-078A302
	(*n* = 602)	(*n* = 300)	(*n* = 302)
Gender, *n* (%)			
Female	410 (68.1)	201 (67.0)	209 (69.2)
Male	192 (31.9)	99 (33.0)	93 (30.8)
Age (years), mean (SD)	58.6 (14.5)	57.1 (15.6)	60.0 (13.2)
ISI score, *n* (%)			
<16	113 (18.8)	63 (21.0)	50 (16.6)
16–21	317 (52.7)	160 (53.3)	157 (52.0)
22–28	170 (28.3)	76 (25.3)	94 (31.2)
Missing data	2 (0.3)	1 (0.3)	1 (0.3)
Mini Mental State Examination score, *n* (%)			
25–27	26 (5.8)	16 (7.5)	10 (4.2)
28–30	426 (94.2)	198 (92.5)	228 (95.8)
Missing data	150 (24.9)	86 (28.7)	64 (21.2)
Body mass index (kg/m^2^), mean (SD)	26.6 (4.4)	26.4 (4.2)	26.7 (4.5)
Health literacy, *n* (%)*			
Adequate	599 (99.5)	298 (99.3)	301 (99.7)
Inadequate	3 (0.5)	2 (0.7)	1 (0.3)
Health numeracy, *n* (%)^†^			
Adequate	527 (87.5)	257 (85.7)	270 (89.4)
Inadequate	75 (12.5)	43 (14.3)	32 (10.6)

ISI, Insomnia Severity Index; SD, standard deviation.

*Health literacy was defined as inadequate if score ≤2 and adequate if >2.

^†^Health numeracy was defined as inadequate if numeracy score ≤2, adequate if >2.

### DCE internal validity

Compared to other health DCEs [36], most patients passed the dominance test (95%) and made consistent choices in the repeated choice tasks (79%). When making choices in the DCE, all patients considered more than one attribute, and 99% considered all alternatives (Supplementary Table S1). In line with recommendations, patients were not excluded from the analysis based on results of internal validity tests to avoid the introduction of selection bias [44].

### Patient preferences

**Figure 3.**
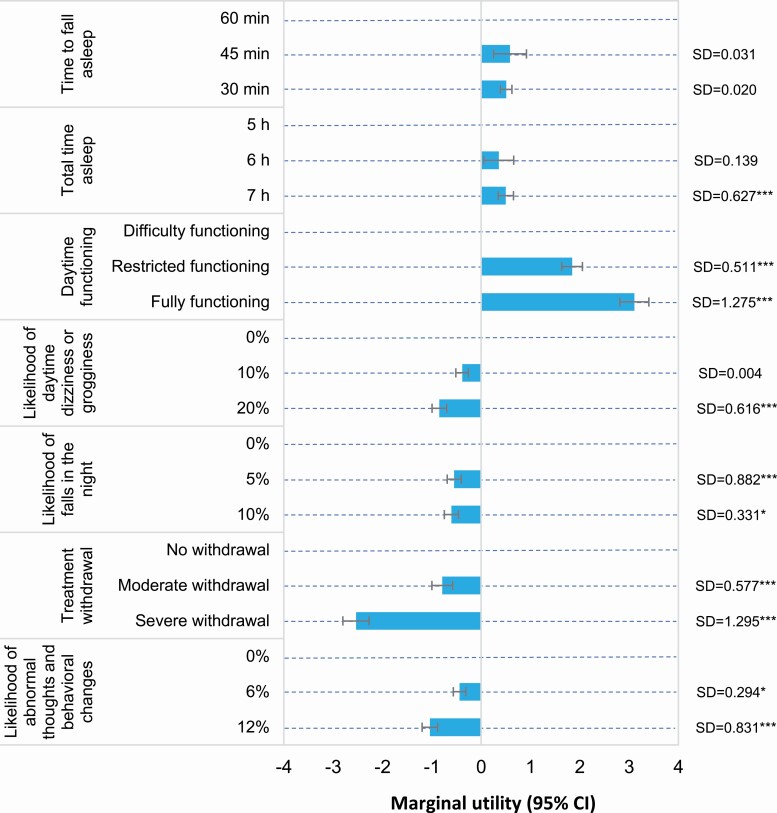
Marginal utilities that capture the effect of changes in attributes on preferences. The estimates are the effect of changes in attributes on preferences (i.e. utility). Positive effects contribute to a treatment being preferred and negative effects reduce the attractiveness of a treatment. A significant standard deviation (SD) denotes the presence of preference heterogeneity. Log-likelihood at convergence = ‐3684. Bayesian information criterion = 7627. Marginal utility of alternative specific constant = 0.235 (*p* < .001). CI, confidence interval; SD, standard deviation. ***p < .001, *p < .05.

The main mixed logit model had a good data fit (adjusted McFadden Pseudo *R*^2^ = 0.258) (Figure 3 and Supplementary Table S2). All attributes significantly affected patients’ preferences (*p* < .001 for all mean estimates). Preference heterogeneity was significant for all attributes except time to fall asleep, indicating that preferences differed between patients.

Daytime functioning (RAI = 33.7%) and withdrawal symptoms (RAI = 27.5%), followed by the likelihood of abnormal thoughts and behavioral changes (RAI = 11.3%) were the most important drivers of treatment preferences (Supplementary Figure S2). Less important were the likelihood of daytime dizziness or grogginess (RAI = 9.2%), likelihood of falls in the night (RAI = 6.5%), time to fall asleep (RAI = 6.4%), and total time asleep (RAI = 5.4%). Improvements in daytime functioning were six times more important than improvements in total time asleep, five times more important than improvements in sleep onset, and three times more important than reductions in the likelihood of abnormal thoughts and behavioral changes. The risks of falls in the night, abnormal thoughts, and behavioral changes, and daytime dizziness/grogginess combined (joint RAI = 27.0%) had similar importance to patients as withdrawal symptoms (RAI = 27.5%).

### Trade-offs between treatment attributes

**Figure 4.**
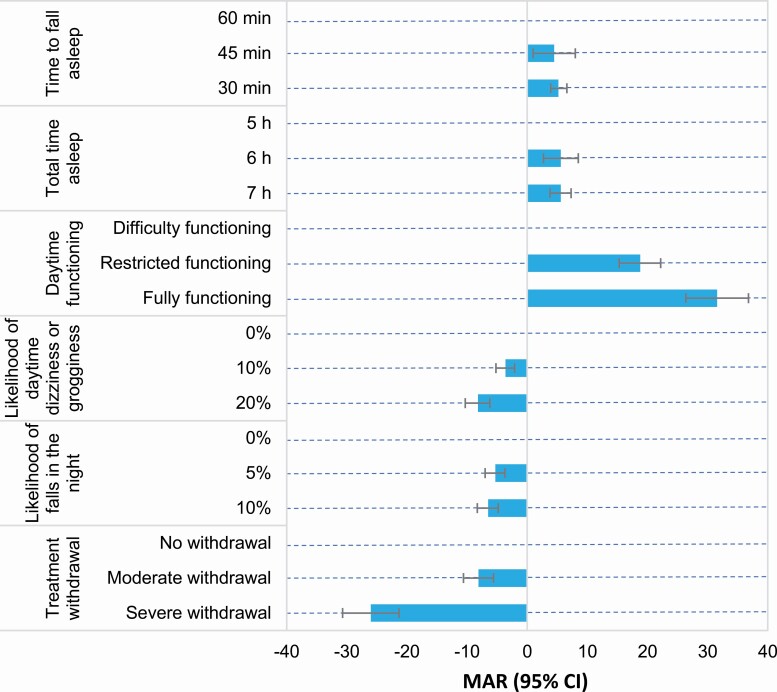
MAR of abnormal thoughts and behavioral changes. MAR normalizes the impact of changes in attributes on preferences using risk equivalences as a common and comparable unit of measurement. CI, confidence interval; MAR, maximum acceptable risk of abnormal thoughts and behavioral changes.

Willingness to make trade-offs between treatment attributes was measured as the additional risk of abnormal thoughts and behaviors that patients were willing to accept to make a change (Figure 4 and Supplementary Table S3). The linear-coded mixed logit model used to obtain MAR estimates is shown in Supplementary Table S4.

Patients were most willing to accept an additional risk of abnormal thoughts and behaviors to improve functioning, including an additional 31.6% risk (*p* < .001) to improve from difficulty functioning to fully functioning, an additional 18.8% risk (*p* < .001) to improve from difficulty functioning to restricted functioning, and an additional 12.8% risk (*p* < .001) to improve from restricted to fully functioning. Patients were also willing to accept an additional risk of abnormal thoughts and behaviors in exchange for a more rapid sleep onset, a longer total time asleep, a reduced risk of daytime dizziness/grogginess, or a reduced risk of falls in the night. For example, they would accept an additional 5.2% risk of abnormal thoughts and behaviors (*p* < .001) to improve sleep onset from 60 to 30 min and an additional 5.6% risk to increase their total time asleep from 5 to 6 h (*p* < .001) or from 5 to 7 h (*p* < .001). Further, they were willing to be compensated with a reduced risk of abnormal thoughts to accept an increased risk of daytime grogginess (e.g. 8.2% decrease to accept a 20% risk of daytime dizziness/grogginess; *p* < .001), an increased risk in falls in the night (e.g. 6.5% decrease to accept a 10% risk of fall in the night; *p* < .001), or worsened daytime functioning (e.g. 26.0% decrease to accept severe withdrawal symptoms; *p* < .001).

Patients were also willing to accept complex trade-offs between multiple attributes, such as a combination of moderate withdrawal effects, a 5% risk of falls at night, and a 10% risk of daytime dizziness/grogginess if their total time asleep increased from 5 to 6 h and their daytime functioning improved from restricted to fully functioning (*p* < .001).

### Differences in preferences between subgroups of patients

Overall, preferences were similar between the two clinical trials (Supplementary Table S5), before treatment (visit 4) and at the end of treatment (visit 8) (Supplementary Table S6), between age groups (18–45 vs. 45–64 vs. ≥ 65 years; Supplemental Table S7), by gender (male vs. female; Supplementary Table S8), and by severity of insomnia at baseline (ISI score < 22 vs. 22–28; Supplementary Table S9). However, some significant differences were found: an increase in the risk of daytime dizziness or grogginess from 0 to 20% was less important to participants at visit 8 than at visit 4; severe withdrawal symptoms was less important to participants aged ≥65 years than to those aged 18–44 years; and risk of daytime dizziness or grogginess was less important to male than female participants. Also, taking 45 min to fall asleep instead of 60 min, improving daytime functioning from difficulty functioning to restricted functioning, and increasing the risk of daytime dizziness or grogginess from 0 to 20% were more important to participants with severe insomnia (ISI score ≥ 22) than to those with mild-to-moderate insomnia (ISI score < 22).

## Discussion

How patients with insomnia view the benefits and risks of different pharmacological treatments has been largely unexplored. This study showed that the preferences of patients with insomnia are strongly driven by their desire to improve daytime functioning and to avoid medication withdrawal effects. This supports proposals that daytime functioning should be considered as an endpoint when evaluating the efficacy of insomnia treatments [45–48]. Beyond implications for clinical research, the findings can also help inform guidelines and discussions between physicians and patients about medication decisions. For example, patients’ concerns about withdrawal symptoms suggested that highlighting differences in the potential for addiction and the ability to stop a treatment are important when describing the different treatment options to patients. Further, the high importance that patients in this study placed on functioning suggests that, in practice, continued monitoring of the effect of treatments on functioning and on the quality of sleep may be needed. This could take a qualitative approach as a discussion with patients or quantitative approach using instruments such as the Insomnia Daytime Symptoms and Impacts Questionnaire.

As expected, patients also preferred a lower risk of experiencing abnormal thoughts and behaviors, daytime dizziness/grogginess, a lower risk of falls in the night, longer total sleep time, and shorter time to sleep onset. These treatment aspects influenced preferences, but daytime functioning (33.7%) and avoidance of withdrawal symptoms (27.5%) each had greater relative importance than abnormal thoughts and feelings, daytime dizziness or grogginess, and falls in the night combined (combined RAI = 27.0%). Patients were willing to accept severe withdrawal effects in exchange for better daytime functioning and some risk of daytime dizziness/grogginess or of falls in the night in exchange for avoiding moderate withdrawal effects and shorter time to sleep onset. However, this study found significant preference heterogeneity in all attributes except time to fall asleep. The trial visit, age, and gender had little influence on patients’ preferences, but patients with more severe insomnia placed more importance on a shorter time to fall asleep, improved daytime functioning, and a lower risk of dizziness or grogginess. To aid in shared decision-making, physicians treating patients with insomnia should consider these preferences and their heterogeneity when discussing treatment options with patients. Objective measures (time to fall asleep and total time asleep) were of low relative importance, suggesting that patients are most concerned with more self-reported measures of feeling well rested. Given the multiple trade-offs that patients were willing to make between different attributes of insomnia treatments, the findings suggest that, in practice, treatment decisions should reflect a range of treatment features. While in current practice clinicians might prescribe based on matching a drug with a pharmacokinetic profile that would address a patient’s primary sleep complaint, a treatment with slightly lower performance with respect to sleep onset but associated with improvements in daytime functioning might be preferred by patients. Therefore, clinicians should discuss with their patients the various pros and cons of different treatment options as a part of shared decision-making rather than prescribing a medication based on one facet of its pharmacokinetic profile.

These findings add to the limited literature on the treatment preferences of patients with insomnia [18, 49]. A single DCE explored treatment preferences of patients with insomnia for attributes common to pharmacological and non-pharmacological treatments [18]. The study, conducted in 205 patients with self-reported insomnia and an ISI score ≥ 14, investigated the influence of time, onset of action, maintainability of improved sleep, length of treatment, and monthly cost on treatment preferences. The current study was the first to explicitly elicit trade-offs patients are willing to make between benefits and risks of a pharmacological treatment and to consider the risk of specific adverse events of interest.

Performing preference elicitation within a clinical trial, as in this case, is new to preference science, and it has two distinct advantages: preference and clinical data are collected from populations that align with the treatment label, and patients are recruited using rigorous screening criteria. Typically, the populations included in preference elicitation studies differ from those in clinical trials and observational studies, which can complicate later interpretation of patient-centered benefit-risk assessment. Eliciting preferences from patients with insomnia can also be complicated because the disease is heterogeneous and case definitions vary [50], which increases the risk of sampling from a different patient population when preference and clinical data are collected in separate studies.

A further important aspect of the current study was the rigorous process used to select the attributes and levels for the DCE. This included an innovative digital ethnography, in-person concept elicitation interviews, and consideration of relevant information about the design of the phase III trials to ensure that the endpoints could be evaluated. Also, one-on-one cognitive interviews were conducted to test whether patients understood and could complete the survey, and a quantitative pilot study [35] was conducted to identify the relevant ranges of included DCE attributes. Finally, several assessments were included to confirm that the internal validity of the DCE was adequate and similar to comparable studies [36].

Despite the multiple strengths of this study, findings should be interpreted in the light of remaining study limitations. Although preference elicitation during the clinical trials had advantages, it may limit the generalizability of the results because preferences of trial participants may differ from real-world populations. Another potential limitation was that not all trial participants opted to participate in the DCE sub-study, although at more than 600 participants, the sample was substantially larger than the 100–300 participants included in typical DCE studies [51]. The population included in this study appeared representative of the general population of patients with chronic insomnia, based on age, gender, and ISI score [52, 53]. No data were collected on prior experience with sleeping pills. Finally, the data from this study can only be interpreted within the context of pharmacological therapies and the included attributes. Administering the DCE within the clinical trial allows trial data to be interpreted from the patients’ perspective, but the data collected here are not suitable for informing choices between non-medical and medical interventions or for overall treatment uptake decisions.

In conclusion, this study showed that valuation of pharmacological insomnia treatments by patients is mostly driven by improvements in daytime functioning, followed by avoidance of withdrawal symptoms, although other aspects of treatments also influenced their preferences. This supports using daytime functioning as an endpoint when evaluating the efficacy of insomnia treatments and carefully weighing the efficacy of a treatment in improving daytime functioning against its risks when making a treatment recommendation in medical practice. This study also demonstrated how preference data can be collected during a clinical trial, which ensures that preference and clinical data are obtained from the same population and allows the influence of treatment on preferences to be determined.

## Supplementary Material

zsac204_suppl_Supplementary_Material

## Clinical Trial Information

The data was collected in two phase III clinical trials:

• “Study to Assess the Efficacy and Safety of ACT-541468 in Adult and Elderly Subjects With Insomnia Disorder”; NCT: NCT03545191—https://clinicaltrials.gov/ct2/show/NCT03545191; EudraCT: 2017-004642-20—https://www.clinicaltrialsregister.eu/ctr-search/search?query=2017-004642-20
• “Study to Assess the Efficacy and Safety of ACT-541468 in Adult and Elderly Subjects Suffering From Difficulties to Sleep”; NCT NCT03575104—https://clinicaltrials.gov/ct2/show/NCT03575104; EudraCT: 2017-004643-20—https://www.clinicaltrialsregister.eu/ctr-search/search?query=2017-004643-20.
