# Supplementary material for: Preferences of patients for benefits and risks of insomnia medications using data elicited during two phase III clinical trials
Source: Sleep. 2022 Sep 2;45(11):zsac204. doi: 10.1093/sleep/zsac204 (PMC9644122; doi:10.1093/sleep/zsac204)
Supplement: zsac204_suppl_Supplementary_Material [file zsac204_suppl_supplementary_material.pdf]

Supplemental materials for “Treatment preferences of patients with insomnia using data elicited during two phase III clinical trials”

Sebastian Heidenreich [a], Melissa Ross [b], Gin Nie Chua [a], Dalma Seboek Kinter [c], Andrea Phillips-Beyer [d]

[a] Evidera, London, UK

[b] Evidera, Bethesda, MD, USA

[c] Idorsia, Allschwil Switzerland

[d] Innovus Consulting, London, UK

Corresponding author: Andrea Phillips-Beyer, Innovus Consulting Ltd., Bellerive House, 3 Muirfield Crescent, London E14 9SZ, UK. Tel: +44 0203 137 7642. Email: innovusconsultingltd@gmail.com.

Contents

Text S1. Supplemental methods 3

Table S1. Internal Validity Assessments for the DCE 6

Table S2. MXL Model (Dummy Coding) 7

Table S3. MAR of abnormal thoughts and behavioral changes 8

Table S4. MXL Model (Linear Coding) 9

Table S5. Effect of Trial on Preferences 10

Table S6. Effect of Treatment on Preferences 11

Table S7. Effect of Age on Preferences 12

Table S8. Effect of Gender on Preferences 13

Table S9. Effect of Insomnia Severity at Baseline on Preferences 14

Figure S1. Trial Design 15

Figure S2. Relative attribute importance 16

Text S1. Supplemental methods

Eligibility criteria

Inclusion criteria:

- Signed informed consent prior to any study-mandated procedure;
- Male or female aged ≥ 18 years;
- Insomnia disorder according to DSM-5 criteria;
- Insomnia Severity Index score ≥ 15;
- Insufficient sleep quantity as collected subjectively in the sleep diary (ID-078A301 trial) or collected subjectively in the sleep diary and validated objectively by polysomnography (ID-078A302 trial);
- Women of childbearing potential must have a negative and urine pregnancy test and use the contraception scheme up to at least 30 days after last study treatment intake.

Exclusion criteria:

- Body mass index below 18.5 or above 40.0 kg/m^2;
- Any lifetime history of related breathing disorder, periodic limb movement disorder, restless legs syndrome, circadian rhythm disorder, rapid eye movement (REM) behavior disorder, narcolepsy, or apnea/hypopnea;
- Cognitive behavioral therapy (CBT) only allowed if, the treatment started at least 1 month prior to Visit 3 and the subject agrees to continue this CBT throughout the study;
- Self-reported usual daytime napping ≥ 1 hour per day and ≥ 3 days per week;
- Acute or unstable psychiatric conditions diagnosed by the Mini International Neuropsychiatric Interview;
- Mini Mental State Examination (MMSE) score < 25 in subjects ≥ 50 years;
- For female subjects: pregnant, lactating or planning to become pregnant during projected duration of the study;
- History or clinical evidence of any disease or medical condition or treatment, which may put the subject at risk of participation in the study or may interfere with the study assessments.
- Any circumstances or conditions, which, in the opinion of the investigator, may affect the subject's full participation in the study or compliance with the protocol.

Preference elicitation

The PAUSe discrete choice experiment (DCE) data were analyzed within a random utility maximization, which assumes that in every DCE choice task (t), each respondent (n), chooses the alternative (j) that results in the highest utility of all available alternatives.[34-37] Utility was defined as:

$$u(\boldsymbol{x}_{jnt}) = v(\boldsymbol{x}_{jnt}) + \varepsilon_{jnt} \quad (1)$$

where the systematic utility component $v(\boldsymbol{x}_{jnt})$ is a function of the DCE attributes, and ε_{jnt} is an extreme value distributed random error, also referred to as psychometric noise. The error term, ε_{jnt} captures random choice influences above and beyond the included attributes and allows the estimation of the utility function as a logit model. The baseline utility, $v(\boldsymbol{x}_{jnt})$ was defined as:

$$v(\boldsymbol{x}_{jnt}) = \begin{array}{l} +\alpha_j \\ +\beta_{1n}\text{time\_to\_sleep\_45min}_{jnt} \\ +\beta_{2n}\text{time\_to\_sleep\_60min}_{jnt} \\ +\beta_{3n}\text{total\_sleep\_6h}_{jnt} \\ +\beta_{4n}\text{total\_sleep\_7h}_{jnt} \\ +\beta_{5n}\text{funtioning\_restricted}_{jnt} \\ +\beta_{6n}\text{funtioning\_fully}_{jnt} \\ +\beta_{7n}\text{dizziness\_10\%}_{jnt} \\ +\beta_{8n}\text{dizziness\_20\%}_{jnt} \\ +\beta_{9n}\text{abnormal\_thoughts\_and\_behaviors\_6\%}_{jnt} \\ +\beta_{10n}\text{abnormal\_thoughts\_and\_behaviors\_12\%}_{jnt} \\ +\beta_{11n}\text{falls\_5\%}_{jnt} \\ +\beta_{12n}\text{falls\_10\%}_{jnt} \\ +\beta_{13n}\text{withdrawal\_moderate}_{jnt} \\ +\beta_{14n}\text{withdrawal\_severe}_{jnt} \end{array} + \varepsilon_{jnt} \quad (2)$$

where α_j is constant for the left alternative in the DCE, controlling for any left-right bias. The remaining parameters are marginal utilities that are assumed to be randomly distributed in the population with a mean and a standard deviation (SD) to be estimated:

β_{1n} captures preference for 45-min time to sleep relative to 30 min

β_{2n} captures preference for 60-min time to sleep relative to 30-min

β_{3n} captures preference for 6-h total time asleep relative to 5 h

β_{4n} captures preference for 7-h total time asleep relative to 5-h

β_{5n} captures preference for restricted functioning relative to difficulty functioning in the daytime

β_{6n} captures preference for fully functioning relative to difficulty functioning in the daytime

β_{7n} captures preference for 10% risk of daytime dizziness relative to no risk

β_{8n} captures preference for 20% risk of daytime dizziness relative to no risk

β_{9n} captures preference for 6% risk of abnormal thoughts and behavior relative to no risk

β_{10n} captures preference for 12% risk of abnormal thoughts and behavior relative to no risk

β_{11n} captures preference for 5% risk of falls in the night relative to no risk

β_{12n} captures preference for 10% risk of falls in the night relative to no risk

β_{13n} captures preference for moderate withdrawal relative to no withdrawal

β_{14n} captures preference for severe withdrawal relative to no withdrawal

The utility function (equation 2) was estimated as a mixed logit (MXL). The MXL is the most general choice model, and accounts for various types of heterogeneity and correlation in the data.[38] The baseline model included patients from both phase III trials with data collected at visit 4.

Relative Attribute Importance (RAI)

Given the ordinal nature of the marginal utilities (i.e., parameters have an arbitrary scale), RAI scores were used to normalize estimates and facilitate the interpretation of the findings. RAI of an attribute (k) was defined as:

$$RAI_k = \frac{\max\{|\bar{\beta}_k|\}}{\sum_{k\in[1;14]} \max\{|\bar{\beta}_k|\}} * 100 \qquad (3)$$

where $\max\{|\bar{\beta}_k|\}$ is the largest marginal utility of any level of attribute k and, given the dummy coding, is equivalent to the maximum affect that the attribute can have on a treatments' utility. Thus, the RAI can be interpreted as a percentage, and measures the proportion of changes in treatment utility that can be assigned to changes in a particular attribute.

Maximum acceptable Risk (MAR)

To obtain insights into attribute trade-offs, MAR estimates were obtained. MAR measures the value of each attribute level, relative to its reference, in its equivalent level of risk of abnormal thoughts and behavior changes. Thus, MAR is a risk trade-off and expresses how much additional risk of abnormal thoughts and behavior changes patients were willing to accept for changes in other attributes.

To calculate MAR, an additional MXL was estimated with the likelihood of abnormal thoughts and behavior changes being a continuous variable. Thus, only one parameter (ϱ_n) instead of β_{9n} and β_{10n} was estimated. The linearity assumption was tested by fitting a linear function through the estimated mean marginal utilities of the likelihood of abnormal thoughts and behavior changes and accepted for an $R^2 > 0.90$. The observed R^2 was 0.992. MAR was defined as

$$MAR_k = -\frac{\bar{\beta}_k}{\bar{\varrho}} \qquad (4)$$

with $\bar{\varrho}$ being the estimated mean of ϱ_n. To ensure that the distribution of MAR_k has finite moments, ϱ_n was specified to follow a log-normal distribution.[47]

Table S1. Internal Validity Assessments for the DCE

	Overall Sample (N=602)	Trial ID-078A301 (N = 300)	Trial ID-078A302 (N = 302)
Dominance test, n (%)			
Fail	29 (4.8)	15 (5.0)	14 (4.6)
Pass	573 (95.2)	285 (95.0)	288 (95.4)
Repeated choice test, n (%)			
Fail	129 (21.4)	73 (24.3)	56 (18.5)
Pass	473 (78.6)	227 (75.7)	246 (81.5)
Attribute trade-offs, n (%)			
Considered more than one attribute	602 (100.0)	300 (100.0)	302 (100.0)
Consider only one attribute	0 (0.0)	0 (0.0)	0 (0.0)
Serial responder, n (%)			
Chose a mixture of options A and B	596 (99.0)	296 (98.7)	300 (99.3)
Always chose option A	4 (0.7)	3 (1.0)	1 (0.3)
Always choosing B	2 (0.3)	1 (0.3)	1 (0.3)
Time to complete DCE (min), n (%)			
< 3.00	107 (17.8)	65 (21.7)	42 (13.9)
3.00 to 4.59	203 (33.7)	108 (36.0)	95 (31.5)
5.00 to 6.59	130 (21.6)	61 (20.3)	69 (22.8)
7.00 to 9.59	124 (20.6)	52 (17.3)	72 (23.8)
10.00 to 14.59	35 (5.8)	13 (4.3)	22 (7.3)
15.00 to 19.59	3 (0.5)	1 (0.3)	2 (0.7)

Abbreviation: DCE, discrete choice experiment

Table S2. MXL Model (Dummy Coding)

	MXL-Overall Sample (N=602)	
	**Marginal utility**	**SD**
**Attribute**	**Coefficient (SE) [95% CI]**	**Coefficient (SE) [95% CI]**
Left Alternative	0.235*** (0.041) [0.155; 0.315]	-
Time to fall asleep		
30 min	Reference	
45 min	0.587*** (0.170) [0.253; 0.920]	0.031 (0.286) [-0.529; 0.592]
60 min	0.508*** (0.060) [0.391; 0.625]	0.020 (0.259) [-0.488; 0.528]
Total time asleep		
5 h	Reference	
6 h	0.360* (0.154) [0.058; 0.661]	0.139 (0.306) [-0.460; 0.738]
7 h	0.501*** (0.079) [0.346; 0.657]	0.627*** (0.094) [0.442; 0.812]
Daytime functioning		
Difficulty functioning	Reference	
Restricted functioning	1.846*** (0.106) [1.637; 2.054]	0.511*** (0.103) [0.309; 0.712]
Fully functioning	3.108*** (0.150) [2.814; 3.401]	1.275*** (0.115) [1.051; 1.500]
Likelihood of daytime dizziness/grogginess		
0%	Reference	
10%	-0.384*** (0.065) [-0.512; -0.257]	0.004 (0.092) [-0.176; 0.184]
20%	-0.846*** (0.076) [-0.994; -0.698]	0.616*** (0.087) [0.444; 0.787]
Likelihood of falls in the night		
0%	Reference	
5%	-0.545*** (0.072) [-0.687; -0.403]	0.882*** (0.089) [0.708; 1.056]
10%	-0.603*** (0.073) [-0.745; -0.460]	0.331* (0.156) [0.026; 0.636]
Withdrawal symptoms		
No withdrawal	Reference	
Moderate withdrawal	-0.786*** (0.108) [-0.997; -0.575]	0.577*** (0.113) [0.355; 0.799]
Severe withdrawal	-2.537*** (0.136) [-2.803; -2.271]	1.295*** (0.110) [1.079; 1.510]
Likelihood of abnormal thoughts and behavioral changes		
0%	Reference	
6%	-0.436*** (0.065) [-0.563; -0.308]	0.294* (0.147) [0.007; 0.581]
12%	-1.037*** (0.081) [-1.196; -0.879]	0.831*** (0.085) [0.665; 0.997]
Log-likelihood	-3684	
Bayesian information criterion	7627	
Adjusted R^2	0.258	

Abbreviations: CI, confidence interval; MXL, mixed logit model; SD, standard deviation; SE, standard error

*** p<0.001, * p<0.05

Table S3. MAR of abnormal thoughts and behavioral changes

Attribute	MAR<br>Coefficient (SE) [95% CI]
Time to fall asleep	
30 min	Reference
45 min	4.5* (1.8) [1.0; 8.0]
60 min	5.2*** (0.7) [3.9; 6.6]
Total time asleep	
5 h	Reference
6 h	5.6*** (1.5) [2.7; 8.5]
7 h	5.6*** (0.9) [3.8; 7.3]
Daytime functioning	
Difficulty Functioning	Reference
Restricted Functioning	18.8*** (1.7) [15.3; 22.2]
Fully Functioning	31.6*** (2.7) [26.4; 36.8]
Likelihood of daytime dizziness or grogginess	
0%	Reference
10%	-3.6*** (0.8) [-5.2; -2.1]
20%	-8.2*** (1.0) [-10.3; -6.2]
Likelihood of falls in the night	
0%	Reference
5%	-5.3*** (0.8) [-7.0; -3.7]
10%	-6.5*** (0.9) [-8.3; -4.8]
Withdrawal symptoms	
No withdrawal	Reference
Moderate withdrawal	-8.1*** (1.3) [-10.6; -5.6]
Severe withdrawal	-26.0*** (2.4) [-30.7; -21.3]

Abbreviations: CI, confidence interval; MAR, maximum acceptable risk of abnormal thoughts and behavioral changes; SE, standard error
*** p<0.001, * p<0.05.

Table S4. MXL Model (Linear Coding)

	MXL-Overall Sample (N=602)	
	**Marginal Utility**	**SD**
**Attribute**	**Coefficient (SE) [95% CI]**	**Coefficient (SE) [95% CI]**
Left Alternative	0.226*** (0.037) [0.154; 0.298]	- -
Time to fall asleep		
30 min	Reference	
45 min	0.414** (0.154) [0.113; 0.715]	0.066 (0.317) [-0.556; 0.687]
60 min	0.480*** (0.058) [0.366; 0.594]	0.006 (0.204) [-0.394; 0.407]
Total time asleep		
5 h	Reference	
6 h	0.515*** (0.139) [0.243; 0.787]	0.050 (0.655) [-1.234; 1.333]
7 h	0.510*** (0.076) [0.361; 0.659]	0.634*** (0.088) [0.462; 0.807]
Daytime functioning		
Difficulty functioning	Reference	
Restricted functioning	1.725*** (0.094) [1.541; 1.910]	0.445*** (0.104) [0.240; 0.650]
Fully functioning	2.907*** (0.135) [2.642; 3.171]	1.063*** (0.105) [0.857; 1.268]
Likelihood of daytime dizziness/grogginess		
0%	Reference	
10%	-0.335*** (0.062) [-0.457; -0.213]	0.001 (0.091) [-0.177; 0.178]
20%	-0.757*** (0.069) [-0.893; -0.621]	0.567*** (0.088) [0.395; 0.739]
Likelihood of falls in the night		
0%	Reference	
5%	-0.490*** (0.070) [-0.627; -0.353]	0.841*** (0.086) [0.673; 1.010]
10%	-0.601*** (0.069) [-0.737; -0.465]	0.214 (0.181) [-0.141; 0.569]
Withdrawal symptoms		
No withdrawal	Reference	
Moderate withdrawal	-0.746*** (0.101) [-0.944; -0.547]	0.180 (0.426) [-0.656; 1.016]
Severe withdrawal	-2.390*** (0.123) [-2.631; -2.149]	1.263*** (0.103) [1.061; 1.465]
Likelihood of abnormal thoughts and behavioral changes	-0.092*** (0.008) [-0.108; -0.076]	0.150*** (0.028) [0.095; 0.206]
Log-likelihood	-3659	
Bayesian information criterion	7557	
Adjusted R^2	0.264	

Abbreviations: CI, confidence interval; MXL, mixed logit model; SD, standard deviation; SE, standard error

** p<0.01, * p<0.05

Table S5. Effect of Trial on Preferences

Attribute	Trial ID-078A301 (N = 300) Main Effect Coefficient [95% CI]	SD Coefficient [95% CI]	Trial ID-078A302 (N = 302) Marginal Effect Coefficient [95% CI]
Left Alternative	0.237*** [0.156, 0.317]	- -	- -
Time to fall asleep			
30 min	Reference		
45 min	0.654** [0.211, 1.097]	0.048 [-0.773, 0.868]	-0.087 [-0.737, 0.563]
60 min	0.514*** [0.354, 0.673]	0.006 [-0.588, 0.601]	-0.022 [-0.243, 0.200]
Total time asleep			
5 h	Reference		
6 h	0.450* [0.045, 0.855]	0.034 [-0.863, 0.931]	-0.249 [-0.847, 0.348]
7 h	0.557*** [0.342, 0.773]	0.660*** [0.478, 0.843]	-0.118 [-0.422, 0.186]
Daytime functioning			
Difficulty functioning	Reference		
Restricted functioning	1.716*** [1.462, 1.971]	0.498*** [0.288, 0.707]	0.292 [-0.052, 0.637]
Fully functioning	2.894*** [2.544, 3.244]	1.252*** [1.028, 1.475]	0.459* [0.022, 0.897]
Likelihood of daytime dizziness or grogginess			
0%	Reference		
10%	-0.377*** [-0.552, -0.201]	0.016 [-0.166, 0.198]	-0.032 [-0.283, 0.220]
20%	-0.849*** [-1.048; -0.650]	0.601*** [0.429; 0.774]	-0.012 [-0.286; 0.262]
Likelihood of falls in the night			
0%	Reference		
5%	-0.511*** [-0.704; -0.318]	0.878*** [0.701; 1.055]	-0.064 [-0.337; 0.208]
10%	-0.575*** [-0.766; -0.384]	0.388** [0.118; 0.658]	-0.045 [-0.316; 0.225]
Withdrawal symptoms			
No withdrawal	Reference		
Moderate withdrawal	-0.901*** [-1.192; -0.611]	0.519*** [0.273; 0.764]	0.213 [-0.196; 0.622]
Severe withdrawal	-2.241*** [-2.563; -1.920]	1.284*** [1.066; 1.503]	-0.625** [-1.055; -0.195]
Likelihood of abnormal thoughts and behavioral changes			
0%	Reference		
6%	-0.488*** [-0.663; -0.314]	0.341** [0.093; 0.589]	0.135 [-0.117; 0.387]
12%	-0.980*** [-1.187; -0.772]	0.831*** [0.662; 0.999]	-0.09 [-0.375; 0.194]
Log-likelihood		-3675	
Bayesian information criterion		7733	
Adjusted R^2		0.257	

Abbreviations: CI, confidence interval; SD, standard deviation
*p<0.05, **p<0.01, ***p<0.001

Table S6. Effect of Treatment on Preferences

Attribute	Visit 4 (N=602) Main Effect Coefficient [95% CI]	Visit 4 (N=602) SD Coefficient [95% CI]	Visit 8 (N=624) Marginal Effect Coefficient [95% CI]
Left Alternative	0.200*** [0.146, 0.254]	-	-
Time to fall asleep			
30 min	Reference		
45 min	0.660*** [0.339, 0.981]	0.005 [-0.588, 0.597]	-0.241 [-0.683, 0.202]
60 min	0.482*** [0.369, 0.595]	0.191 [-0.096, 0.479]	0.004 [-0.149, 0.157]
Total time asleep			
5 h	Reference		
6 h	0.312* [0.017, 0.606]	0.054 [-0.473, 0.582]	-0.047 [-0.456, 0.363]
7 h	0.507*** [0.356, 0.658]	0.652*** [0.524, 0.779]	-0.030 [-0.237, 0.177]
Daytime functioning			
Difficulty functioning	Reference		
Restricted functioning	1.867*** [1.676, 2.057]	0.380*** [0.214, 0.545]	-0.004 [-0.238, 0.230]
Fully functioning	3.060*** [2.806, 3.315]	1.089*** [0.936, 1.241]	0.047 [-0.241, 0.335]
Likelihood of daytime dizziness or grogginess			
0%	Reference		
10%	-0.388*** [-0.513, -0.263]	0.001 [-0.130, 0.133]	0.099 [-0.074, 0.272]
20%	-0.824*** [-0.962; -0.686]	0.484*** [0.350; 0.618]	0.257** [0.072; 0.442]
Likelihood of falls in the night			
0%	Reference		
5%	-0.538*** [-0.673; -0.402]	0.798*** [0.675; 0.922]	0.103 [-0.081; 0.286]
10%	-0.605*** [-0.744; -0.465]	0.475*** [0.316; 0.634]	0.108 [-0.081; 0.296]
Withdrawal symptoms			
No withdrawal	Reference		
Moderate withdrawal	-0.819*** [-1.022; -0.616]	0.574*** [0.417; 0.732]	0.269 [-0.010; 0.548]
Severe withdrawal	-2.537*** [-2.783; -2.291]	1.435*** [1.278; 1.593]	0.089 [-0.212; 0.389]
Likelihood of abnormal thoughts and behavioral changes			
0%	Reference		
6%	-0.421*** [-0.546; -0.295]	0.287** [0.099; 0.475]	0.040 [-0.133; 0.213]
12%	-1.013*** [-1.162; -0.865]	0.788*** [0.672; 0.904]	0.181 [-0.014; 0.376]
Log-likelihood		-7569	
Bayesian information criterion		15551	
Adjusted R^2		0.254	

Abbreviations: CI, confidence interval; SD, standard deviation
*p<0.05, ***p<0.001

Table S7. Effect of Age on Preferences

Attribute	18-45 years (N=109) Main Effect Coefficient [95% CI]	SD Coefficient [95% CI]	45-64 years (N=179) Marginal Effect Coefficient [95% CI]	≥ 65 years (N=314) Marginal Effect Coefficient [95% CI]
Left Alternative	0.242*** [0.161, 0.323]	-	-	-
Time to fall asleep				
30 min	Reference			
45 min	0.444 [-0.312, 1.201]	0.024 [-0.749, 0.797]	0.036 [-0.937, 1.009]	-0.444 [-0.614; 1.125]
60 min	0.449** [0.173, 0.725]	0.022 [-0.575, 0.620]	0.138 [-0.208, 0.483]	-0.159 [-0.264; 0.360]
Total time asleep				
5 h	Reference			
6 h	0.275 [-0.427, 0.976]	0.028 [-0.792, 0.849]	0.219 [-0.676, 1.115]	-0.413 [-0.760; 0.858]
7 h	0.608** [0.244, 0.972]	0.649*** [0.463, 0.834]	-0.079 [-0.537, 0.379]	-0.213 [-0.562; 0.274]
Daytime functioning				
Difficulty functioning	Reference			
Restricted functioning	1.716*** [1.298, 2.134]	0.485*** [0.271, 0.698]	0.359 [-0.153, 0.872]	-0.236 [-0.394; 0.532]
Fully functioning	3.421*** [2.844, 3.997]	1.236*** [1.012, 1.459]	0.031 [-0.634, 0.696]	-0.31 [-1.183; 0.031]
Likelihood of daytime dizziness or grogginess				
0%	Reference			
10%	-0.384* [-0.691; -0.078]	0.024 [-0.159; 0.207]	0.143 [-0.241; 0.527]	-0.179 [-0.429; 0.272]
20%	-0.796*** [-1.134; -0.458]	0.607*** [0.435; 0.780]	0.124 [-0.297; 0.545]	-0.195 [-0.552; 0.212]
Likelihood of falls in the night				
0%	Reference			
5%	=-0.425* [-0.756; -0.093]	0.885*** [0.707; 1.062]	-0.245 [-0.662; 0.171]	-0.193 [-0.492; 0.264]
10%	-0.619*** [-0.959; -0.280]	0.397** [0.136; 0.658]	0.066 [-0.354; 0.486]	-0.195 [-0.380; 0.384]
Withdrawal symptoms				
No withdrawal	Reference			
Moderate withdrawal	-1.096*** [-1.604; -0.588]	0.522*** [0.281; 0.763]	0.133 [-0.500; 0.765]	-0.293 [-0.132; 1.017]
Severe withdrawal	-3.050*** [-3.612; -2.488]	1.308*** [1.090; 1.526]	0.469 [-0.192; 1.131]	-0.306 [0.055; 1.253]
Likelihood of abnormal thoughts and behavioral changes				
0%	Reference			
6%	-0.640*** [-0.948; -0.332]	0.353** [0.108; 0.597]	0.232 [-0.153; 0.618]	-0.179 [-0.104; 0.599]
12%	-1.159*** [-1.509; -0.809]	0.836*** [0.665; 1.007]	0.062 [-0.373; 0.497]	-0.201 [-0.215; 0.574]
Log-likelihood		-3664		
Bayesian information criterion		7834		
Adjusted R^2		0.257		

Abbreviations: CI, confidence interval; SD, standard deviation
*p<0.05, **p<0.01, ***p<0.001

Table S8. Effect of Gender on Preferences

	Female (N=410)		Male (N=192)
Attribute	Main Effect Coefficient [95% CI]	SD Coefficient [95% CI]	Marginal Effect Coefficient [95% CI]
Left Alternative	0.238*** [0.158, 0.319]	-	-
Time to fall asleep			
30 min	Reference		
45 min	0.594** [0.188, 1.001]	0.041 [-0.729, 0.812]	-0.017 [-0.697, 0.662]
60 min	0.482*** [0.342, 0.623]	0.008 [-0.548, 0.563]	0.084 [-0.152, 0.321]
Total time asleep			
5 h	Reference		
6 h	0.36 [-0.011, 0.731]	0.01 [-0.904, 0.924]	-0.026 [-0.651, 0.599]
7 h	0.507*** [0.316, 0.697]	0.655*** [0.471, 0.839]	0.008 [-0.315, 0.332]
Daytime functioning			
Difficulty functioning	Reference		
Restricted functioning	1.927*** [1.680, 2.173]	0.507*** [0.301, 0.714]	-0.235 [-0.594, 0.125]
Fully functioning	3.173*** [2.839, 3.506]	1.252*** [1.028, 1.477]	-0.166 [-0.630, 0.298]
Likelihood of daytime dizziness or grogginess			
0%	Reference		
10%	-0.478*** [-0.635; -0.322]	0.019 [-0.163; 0.201]	0.293* [0.025; 0.561]
20%	-0.954*** [-1.133; -0.776]	0.605*** [0.432; 0.778]	0.337* [0.043; 0.631]
Likelihood of falls in the night			
0%	Reference		
5%	-0.545*** [-0.716; -0.373]	0.881*** [0.705; 1.058]	-0.014 [-0.304; 0.277]
10%	-0.549*** [-0.718; -0.379]	0.379** [0.106; 0.652]	-0.143 [-0.434; 0.147]
Withdrawal symptoms			
No withdrawal	Reference		
Moderate withdrawal	-0.872*** [-1.127; -0.617]	0.532*** [0.292; 0.771]	0.242 [-0.198; 0.682]
Severe withdrawal	-2.634*** [-2.949; -2.319]	1.306*** [1.088; 1.524]	0.279 [-0.173; 0.730]
Likelihood of abnormal thoughts and behavioral changes			
0%	Reference		
6%	-0.432*** [-0.587; -0.276]	0.339** [0.085; 0.594]	-0.016 [-0.285; 0.253]
12%	-1.090*** [-1.279; -0.901]	0.828*** [0.658; 0.998]	0.163 [-0.140; 0.466]
Log-likelihood	-3677		
Bayesian information criterion	7737		
Adjusted R^2	0.257		

Abbreviations: CI, confidence interval; Coeff, coefficient; SD, standard deviation
*p<0.05, **p<0.01, ***p<0.001

Table S9. Effect of Insomnia Severity at Baseline on Preferences

Attribute	Mild-moderate (ISI < 22) (N=430) Main Effect Coefficient [95% CI]	Mild-moderate (ISI < 22) (N=430) SD Coefficient [95% CI]	Severe (ISI = 22-28) (N=170) Marginal Effect Coefficient [95% CI]
Left Alternative	0.237*** [0.156, 0.319]	-	-
Time to fall asleep			
30 min	Reference		
45 min	0.829*** [0.420, 1.238]	0.037 [-0.528, 0.602]	-0.802* [-1.506,-0.097]
60 min	0.511*** [0.373, 0.649]	0.057 [-0.623, 0.737]	0.016 [-0.234,0.266]
Total time asleep			
5 h	Reference		
6 h	0.195 [-0.169, 0.559]	0.093 [-0.661, 0.847]	0.505 [-0.152, 1.161]
7 h	0.459*** [0.273, 0.645]	0.660*** [0.472, 0.847]	0.183 [-0.158, 0.524]
Daytime functioning			
Difficulty Functioning	Reference		
Restricted Functioning	2.015*** [1.762, 2.269]	0.480*** [0.262, 0.697]	-0.493** [-0.859, -0.127]
Fully Functioning	3.287*** [2.938, 3.636]	1.276*** [1.047, 1.505]	-0.437 [-0.915, 0.041]
Likelihood of daytime dizziness or grogginess			
0%	Reference		
10%	-0.427*** [-0.581; -0.274]	0.002 [-0.183; 0.187]	0.147 [-0.132; 0.426]
20%	-0.947*** [-1.126; -0.768]	0.628*** [0.450; 0.805]	0.334* [0.025; 0.643]
Likelihood of falls in the night			
0%	Reference		
5%	-0.573*** [-0.743; -0.403]	0.899*** [0.722; 1.075]	0.049 [-0.258; 0.355]
10%	-0.685*** [-0.856; -0.513]	0.417*** [0.170; 0.663]	0.278 [-0.022; 0.578]
Withdrawal symptoms			
No withdrawal	Reference		
Moderate withdrawal	-0.791*** [-1.043; -0.539]	0.572*** [0.325; 0.819]	-0.01 [-0.469; 0.449]
Severe withdrawal	-2.574*** [-2.891; -2.258]	1.330*** [1.103; 1.557]	-0.018 [-0.485; 0.450]
Likelihood of abnormal thoughts and behavioral changes			
0%	Reference		
6%	-0.379*** [-0.533; -0.226]	0.354** [0.112; 0.597]	-0.186 [-0.469; 0.096]
12%	-1.023*** [-1.208; -0.838]	0.848*** [0.675; 1.022]	-0.103 [-0.425; 0.220]
Log-likelihood	-3664		
Bayesian information criterion	7710		
Adjusted R^2	0.26		

Abbreviations: CI, confidence interval; SD, standard deviation
*p<0.05, **p<0.01, ***p<0.001

Figure S1. Trial Design

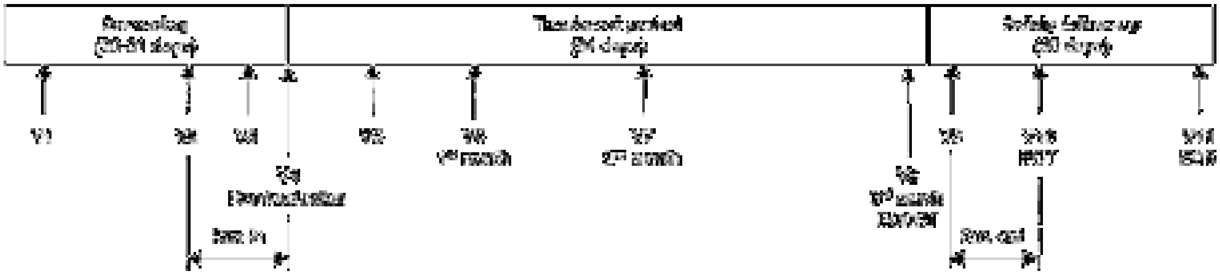

Abbreviations: EODBT, end of double-blind treatment; EOS, end of study; EOT, end of treatment; V, visit

Figure S2. Relative attribute importance

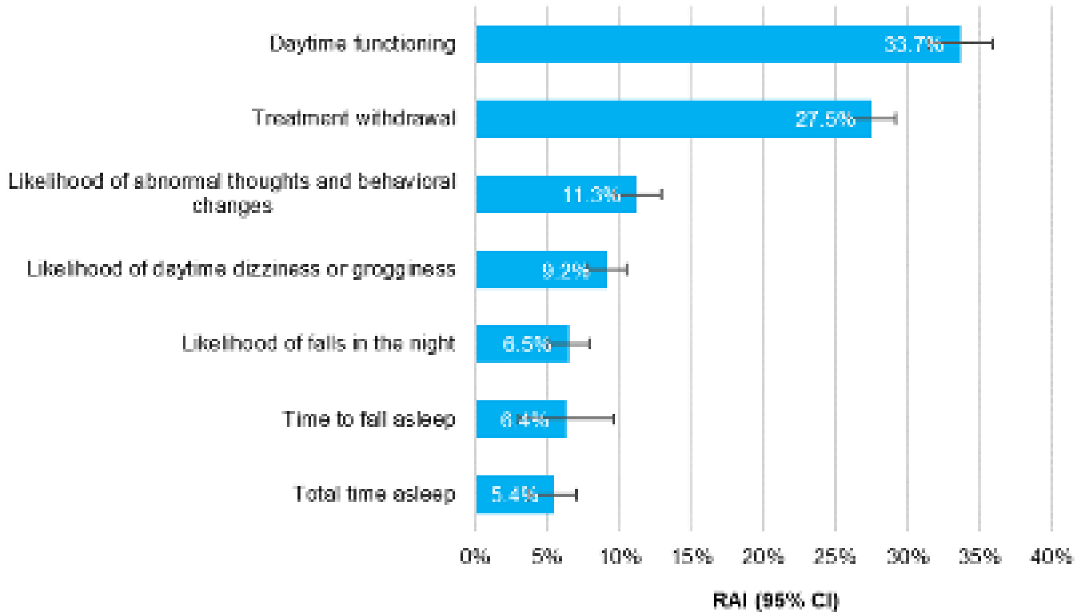

Abbreviations: CI, confidence interval; RAI, relative attribute importance
